# Repeated evolution of similar phenotypes: Integrating comparative methods with developmental pathways

**DOI:** 10.1590/1678-4685-GMB-2022-0384

**Published:** 2023-07-14

**Authors:** Anieli Guirro Pereira, Tiana Kohlsdorf

**Affiliations:** 1 Universidade de São Paulo, Faculdade de Filosofia, Ciências e Letras de Ribeirão Preto (FFCLRP), Departamento de Biologia, Ribeirão Preto, SP, Brazil.

**Keywords:** Repeated evolution, recurrent phenotypes, comparative methods, developmental pathways

## Abstract

Repeated phenotypes, often referred to as ‘homoplasies’ in cladistic analyses, may evolve through changes in developmental processes. Genetic bases of recurrent evolution gained attention and have been studied in the past years using approaches that combine modern analytical phylogenetic tools with the stunning assemblage of new information on developmental mechanisms. In this review, we evaluated the topic under an integrated perspective, revisiting the classical definitions of convergence and parallelism and detailing comparative methods used to evaluate evolution of repeated phenotypes, which include phylogenetic inference, estimates of evolutionary rates and reconstruction of ancestral states. We provide examples to illustrate how a given methodological approach can be used to identify evolutionary patterns and evaluate developmental mechanisms associated with the intermittent expression of a given trait along the phylogeny. Finally, we address why repeated trait loss challenges strict definitions of convergence and parallelism, discussing how changes in developmental pathways might explain the high frequency of repeated trait loss in specific lineages.

## Introduction

Similar phenotypes may emerge several times along the evolutionary history of a given lineage, characterizing phylogenetically-discontinuous traits that are often referred to as ‘homoplasies’ in cladistic analyses (see West-Eberhard, 2003; Wake et al., 2011; Orgogozo, 2015). The intermittent expression of a given trait along an evolutionary trajectory is developmentally feasible because regulatory changes may modulate genetic pathways and also turn on and off the signaling cascades related to the establishment of that phenotype (West-Eberhard, 2003). Genetic mechanisms involved in the repeated evolution of specific traits have puzzled researchers for decades (e.g. Hunt et al., 1998; Shi and Yokoyama, 2003; Schluter et al., 2004; Rosenblum et al., 2010; Davies et al., 2012; Guerreiro et al., 2013; Projecto-Garcia et al., 2013; Liu et al., 2014; Nery et al., 2016; Mohammadi et al., 2016; Hu et al., 2017; Liu et al., 2022). Recent advances in modern analytical phylogenetic tools and the stunning assemblage of new information on developmental mechanisms in the past years enable us to evaluate the topic under an integrated perspective and also to revisit major concepts and classical examples of phenotypic recurrence in nature. We start this review by reassessing the classical definitions of convergence and parallelism at different biological levels. Then, we detail the principal comparative methods used to evaluate repeated evolution of similar phenotypes, focusing on phylogenetic inference, estimates of evolutionary rates and reconstruction of ancestral states. Together with the synthetic presentation of each method, we provide a few examples to illustrate how that methodological approach can be used to identify evolution patterns and evaluate developmental mechanisms associated with the intermittent expression of a given trait along the phylogeny. Finally, we discuss why repeated trait loss challenges strict definitions of convergence and parallelism, and address how changes in developmental pathways might explain the high frequency of repeated trait loss in specific lineages. Across this discussion, we adopt the expression ‘recurrent phenotypes’ to refer to similar traits that emerged several times along a given phylogeny regardless of the genetic mechanism underlying the evolution of such similarities, so that the term per se does not imply a distinction between parallelism or convergence at the phenotypic level (as further explained, see also West-Eberhard, 2003 for an extensive discussion on ‘recurrence’).

### Revising concepts: Convergence and parallelism

**Figure 1 -:**
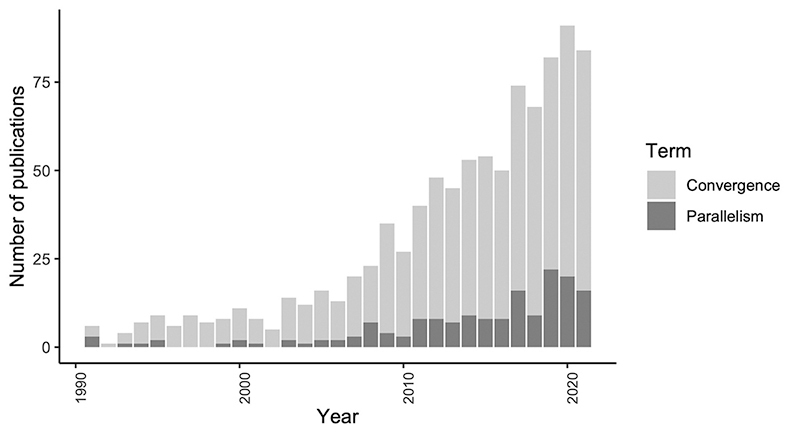
Number of publications with the terms “convergence/convergent evolution” (light gray) and “parallelism/parallel evolution” (dark gray) from 1990 to 2021. Data retrieved from Web of Science (https://www.webofscience.com/).

The extensive interest on how similar phenotypes repeatedly evolved in nature has motivated researchers from different fields to intensively investigate the mechanisms associated to these similarity patterns and to propose concepts delimiting the processes that explain recurrent phenotypes. Two concepts - convergence and parallelism - have appeared with increasing frequency in evolutionary studies along the past three decades (Figure 1), and are addressed in this section. Equivalent selective pressures are often claimed to be a possible explanation for the recurrent evolution of similar phenotypes among phylogenetically-distant lineages (see Wake, 1991). Given that the same phenotype might result from different genetic trajectories (a concept known as ‘many-to-one’ mapping of genotype to phenotype), the repeated evolution of similar phenotypes turns into an even more interesting event (Storz, 2016). Accordingly, the concepts of convergence and parallelism ultimately focus on how similar are the mechanisms underlying a recurrent phenotype.

**Figure 2 -:**
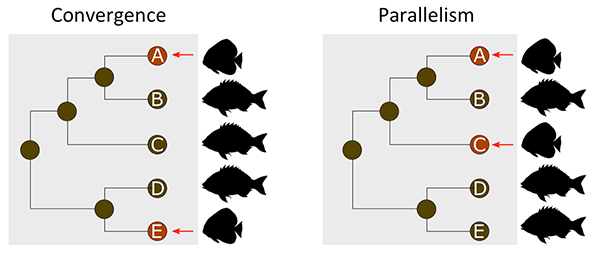
Application of the terms “convergence” and “parallelism” at the phenotypic level was originally based on the phylogenetic distance among lineages that evolved similar phenotypes.

At the phenotypic level, evolutionary similarities observed among different lineages (here termed ‘recurrent phenotypes’) have been classically defined as *parallel* or *convergent* evolution (see Scotland, 2011; Rosenblum *et al.*, 2014) based initially on the distances among taxa. Specifically, similar phenotypes among closely related lineages agree with the definition of *parallelism*, while those among distantly related taxa would correspond to *convergence* (Figure 2). The criterion for differentiating ‘distance’ among lineages, however, may be vague (see Davis and Heywood, 1963; Conte *et al.*, 2012; Rosenblum *et al.*, 2014). Other studies provided alternative definitions for both terms (reviewed in Gompel and Prud’homme, 2009; Wake *et al.*, 2011), until completely removing the term *parallelism* from the classification of evolutionary similarities at the phenotypic level (see Arendt and Reznick, 2008). In this review, we opted for not distinguishing *convergent* and *parallel* evolution at the phenotypic level; instead, we adopt the term ‘recurrent phenotypes’ and untangle this discussion from the main focus of our review, which are the genetic mechanisms underlying evolution of phenotypic similarities among different lineages. 

**Figure 3 -:**
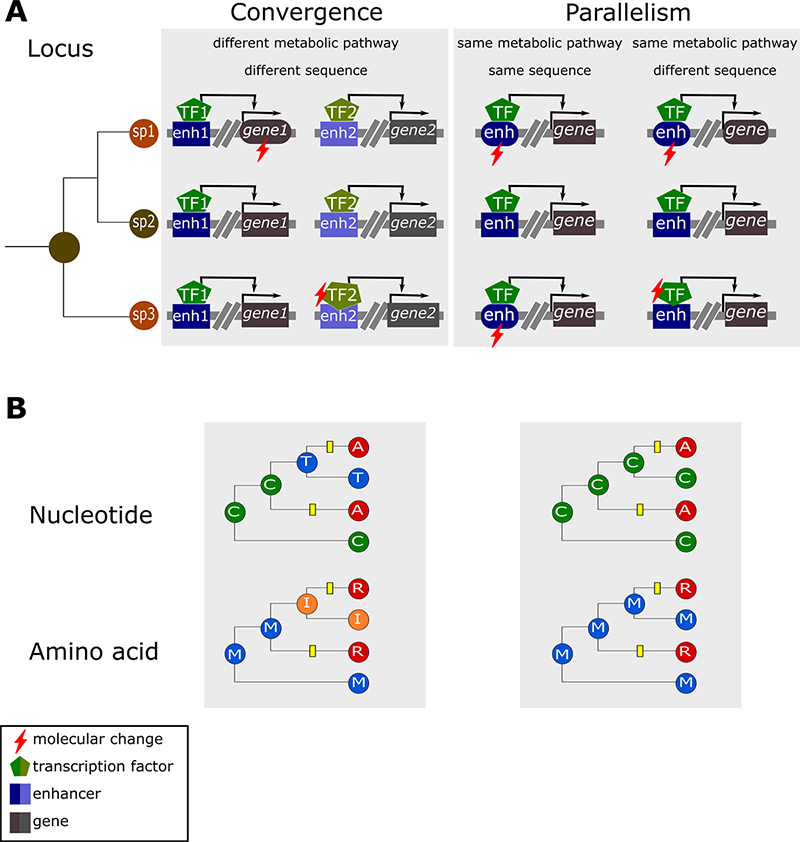
The terms “convergence” and “parallelism” are used to describe the genetic basis of recurrent phenotypes at two different levels: (A) locus and (B) nucleotide or amino acid sites. (A) At the locus level, species 1 (sp1) and species 3 (sp3) share a recurrent phenotype. In the box at the left (‘Convergence’), the red ray indicates the molecular basis (*gene1* and *TF2*, respectively) associated with the recurrent phenotype in sp1 and sp3, illustrating a case of molecular convergence in which genetic changes in the species reside at different signaling pathways. In the box at the right (‘Parallelism’), the example along the column ‘same metabolic pathway/same sequence’ illustrates a genetic basis of the recurrent phenotype in sp1 and sp3 settled at the enhancer (red ray), while that the column ‘same metabolic pathway/different sequence’ illustrates a case where genetic changes in sp1 and sp3 locate at different components of the same signaling pathway (red rays at the *gene* and the *TF*, respectively). (B) Site substitutions from different ancestral nucleotides or amino acids represent a convergence (left), while those resulting from the same trajectory are defined as a parallelism (right)

The molecular processes associated with recurrent phenotypes are often unknown, and several studies aim to elucidate whether repeated evolution is usually settled on the same or in different developmental pathways (as further discussed in this review). We can evaluate the molecular bases of phenotypic recurrence at two levels: 1) the *locus level*, which concerns the molecules (e.g. DNA sequence or protein) as a whole; and 2) the *site level*, which considers each site (e.g. nucleotide or amino acid) independently. At the *locus level*, recurrent phenotypes from trait changes involving different metabolic pathways are defined as *convergence* (see box in the left at Figure 3a), while those involving changes in the same metabolic pathway are referred to as *parallelism* (see box in the right at Figure 3A). Cases interpreted as *parallelism* can be also evaluated regarding whether the identified changes reside in the same genome regions or not (see Figure 3A). At the site level, two or more lineages can independently have the same nucleotide or amino acid at the same position (Figure 3B). When the ancestral basis or the ancestral amino acid is the same for both lineages, it is considered a *parallel substitution*. In the case of different origins, these substitutions are referred to as *convergent substitutions* (Storz, 2016).

Molecular patterns can be also categorized based on their location within the genome. In this case, changes in protein-coding regions are often regarded as ‘genetic’, while changes in non-coding genomic loci are frequently referred to as regulatory or epigenomic. For instance, both have potential effects on the phenotype - the former by directly modifying the protein sequence and structure, and the latter by influencing gene expression (Bulger and Groudine, 2011; Meddens *et al.*, 2019).

### Crosstalk between convergence-parallelism and regulatory networks-gene interactions

As aforementioned, molecular patterns at the *locus level* associated with recurrent phenotypes are usually defined as *parallelism* when involving the same sequences, and as *convergence* when related to different sequences. The comparison of orthologous sequences or proteins has been a central point for several studies that evaluated molecular bases of recurrent phenotypes (e.g. Rosenblum *et al.* 2010; Davies *et al.*, 2012; Guerreiro *et al.*, 2013; Projecto-Garcia *et al.*, 2013; Liu *et al.*, 2014; Mohammadi *et al.*, 2016; Nery *et al.*, 2016; Hu *et al.*, 2017; Liu *et al.*, 2022). Nonetheless, genes are part of regulatory networks, interacting with cis-regulatory elements (such as enhancers and promoters) and transcription factors that control the expression of one gene or a group of genes (Babu *et al.*, 2004; Wagner and Lynch, 2008; Voordeckers *et al.*, 2015). A greater number of sequences working together, as in complex regulatory networks, might confer flexibility to developmental interactions and eventually facilitate repeated evolution of similar phenotypes in different lineages (see Orr, 2005; Rosenblum *et al.,* 2014; Yeaman *et al.*, 2018; Pereira *et al.*, 2022). 

Pleiotropy is also an important topic to be considered in discussions regarding the molecular bases of recurrent phenotypes and associated regulatory pathways. Several genes are pleiotropic, which means that a given gene is involved in the establishment of different phenotypic traits (Lobo, 2008). Changes in that gene, therefore, likely affect several processes simultaneously. In highly pleiotropic genes, changes in cis-regulatory elements might be a powerful tool in evolution because the modular architecture of these regions enable that changes affecting gene expression in specific tissues or cells and also modifying developmental times of specific structures do not compromise other phenotypic traits (Prud’homme *et al.*, 2006; Monteiro and Podlaha, 2009; Feigin *et al.*, 2019, Morris *et al.*, 2020). 

Despite several studies focusing on cis-regulatory convergent evolution (e.g. Booker *et al.*, 2016; Kvon *et al.*, 2016; Partha *et al.*, 2017; Tollis *et al.*, 2018; Feigin *et al.*, 2019; Sackton *et al.*, 2019), some questions remain central to this discussion. Do different changes in the same regulatory pathway challenge strict definitions of convergence and parallelism? After all, when changes occur in different sequences that are involved in the same regulatory network, but also associated with other developmental pathways, shall we classify them as *convergence*, or *parallelism*?

## Comparative methods: Molecular associations of recurrent phenotypes

In this section, we focus on phylogenetic comparative methods (PCMs) based on a phylogenetic inference that are frequently used to address the molecular bases of recurrent phenotypes. Phylogeny and ancestral character reconstructions are essential to evaluate repeated evolution of a given phenotype among different lineages (see Speed and Arbuckle, 2017 for a review in methods of studies addressing recurrent phenotypes). Phylogenetic inferences aim to recover information from the topology (=the relative branching order) and branch lengths (=evolutionary distance or probability of character change) related to a given group (Baum and Smith, 2013). Several methods have been developed for phylogenetic inference (e.g., distance and statistical or probabilistic methods), and this step is considered essential to evaluate evolutionary patterns of recurrent phenotypes (Garland *et al.*, 2005). Probabilistic methods are represented by the maximum likelihood (Felsenstein, 1981, 1985) and Bayesian (Rannala and Yang, 1996; Mau *et al.*, 1999) approaches. 

The increasing availability of genomic data makes it possible to perform a comprehensive search for signatures of similarities in a genomic scale (Speed and Arbuckle, 2017). Several studies use tools for a genomic search (e.g. Thomas and Hahn, 2015; Chikina *et al.*, 2016; Hu *et al.*, 2017; Sackton *et al.*, 2019), while others focus on certain genes or specific regulatory pathways already known to be related with the studied phenotype (e.g. Mohammadi *et al.*, 2016; Pereira *et al.*, 2022). Significant progress in the fields of comparative genomics and functional genomics recently provided a deep understanding of regulatory mechanisms likely involved in these evolutionary processes (Lamichhaney *et al.*, 2019).

### Gene/site tree and species tree incongruence

The phylogeny inference based on one genetic locus results in a gene tree, or *genealogy.* This approach contrasts with that used for a species tree, which contains several, if not all, gene trees (Maddison, 1997). In practice, the species tree based on molecular data can be built using a group of concatenated genes [supermatrix approach] or as a summary of dozens of gene trees [multispecies coalescent approach] (Rannala *et al.*, 2020). Some of the software used to perform these analyses are synthesized at Table 1. Incongruence between the genealogy and a species tree can result from diverse biological factors, including incomplete lineage sorting [ILS], introgression, and lateral gene transfer (see Maddison, 1997). These factors are called *hemiplasy*, a term used to define a pattern similar to homoplasy but produced by a non-homoplasy event, which may result in an apparent similarity in the genealogy and also affect reconstructions of the ancestral sequence (Avise and Robinson, 2008; Mendes *et al.*, 2016). 

**Table 1 -:** Comparative analyses used to evaluate convergent and parallel evolution, with most used software and associated references.

Analysis		Softwares		References
Phylogenetic Tree Inference (Topo) Analysis of Evolutionary Rates (BL) Concordance factor of genes (gCFs) Concordance factor of sites (sCFs) Phylogenetic signal (PS)	Topo and BL	RaxML		Stamatakis, 2006
		IQTree		Nguyen *et al.*, 2015; Minh *et al.*, 2020b
		MrBayes		Ronquist & Huelsenbeck, 2003
		BEAST		Drummond & Rambaut, 2007; Bouckaert *et al.*, 2014
	BL	aaML	PAML	Yang, 2007
	gCFs	IQTree		Minh *et al.*, 2020a
	sCFs	IQTree		Mo *et al.*, 2023
	PS	SH-test	CONSEL	Shimodaira and Hasegawa, 2001
Correlations between morphotypes and sequence rates		Forward Genomics		Hiller *et al.*, 2012; Prudent *et al.*, 2016
		TraitRateProp		Levy *et al.*, 2017
		TraitRELAX		Halabi *et al.*, 2020
		RERconverge		Kowalczyk *et al.*, 2019
		Coevol		Lartillot & Poujol, 2011
		PhyloAcc		Hu *et al.*, 2019
		PhyloAcc-GT		Yan *et al.*, 2022
Ancestral state reconstruction		make.simmap/phytools	R	Revell, 2012
		baseML or codeML	PAML	Yang, 2007
Selection tests (Branch/Clade Model)		codeML	PAML	Yang, 2007
		aBSREL	HyPhy	Smith *et al.*, 2015
		BUSTED		Murrell *et al.*, 2015
		RELAX		Wertheim *et al.*, 2015
Selection Tests (Branch-site Model)		codeML	PAML	Yang, 2007
		FEL	HyPhy	Pond *et al.*, 2005
		FUBAR		Murrell *et al.*, 2013
		MEME		Murrell *et al.*, 2012
		SLAC		Pond *et al.*, 2005

Incongruence between topologies may also represent genetic convergence or parallelism (homoplasy) and, in this case, the comparison of gene and species trees represents an effective approach, for both coding and regulatory sequences. As phylogenetic analyses compare site-by-site similarities, convergence or parallelism in one or more sites (as illustrated in Figure 3) may erroneously group species, possibly influencing the phylogenetic inference analyses and causing a genetic tree discordance (i.e. clustering phylogenetically unrelated species in the gene tree), which is also known as *phylogenetic incongruence*. Therefore, the comparison between a gene topology and the most-accepted species tree is a tool used to detect possible effects of molecular similarity (Davies *et al.*, 2012). Some methods have been developed to assist identification of the proportion of genes (gene support frequency or gene concordance factor) and sites (site concordance factor) that align with a given species tree (Ané *et al.*, 2007; Minh *et al.*, 2020a; Mo *et al.*, 2023), as synthesized in Table 1.

It is worth noting that this approach detects similarity but does not distinguish convergence from parallelism. Subsequent tests estimating the phylogenetic signal can provide a statistical value of how much the alternative topology (*gene tree*) is supported given the expected species phylogeny (see Blomberg *et al.* 2003; Münkemüller *et al.* 2012). A more quantitative approach is, however, necessary to estimate evolutionary parameters and test competing hypotheses (Ansari and Didelot, 2016).

### An example of phylogenetic inference: repeated evolution of laryngeal echolocation in bats

A topic that exemplifies the application of phylogenetic tree inference is the repeated evolution of echolocation among bats. Echolocation is a biological sonar that evolved independently in lineages as distant as bats and whales (Shen *et al.*, 2012; Liu *et al.*, 2014; Thomas and Hahn, 2015). Within Chiroptera (bats), this phenotype is observed in two non-related lineages: the suborder Yangochiroptera and the superfamily Rhinolophoidea (suborder Yinpterochiroptera). In addition to the superfamily Rhinolophoidea, Yinpterochiroptera also includes the Pteropodidae family of non-echolocating Old World fruit bats (Liu *et al.*, 2014). As specialized hearing co-evolves with echolocation, two genes (*Tmc1* and *Pjvk*) associated with nonsyndromic hearing loss in mammals are particularly interesting to understand the evolution of echolocation among bats (Vater and Kössl, 2004; Xu *et al.*, 2013). Phylogenetic inference estimating gene trees for *Tmc1* and *Pjvk* erroneously group laryngeal echolocating bat lineages in a monophyletic clade (see Davies *et al.*, 2012), suggesting molecular similarity of these genes among groups. Subsequent studies (see Liu *et al.*, 2022) revisited the topic and found evidence for a single origin of laryngeal echolocation in bats and an eventual loss in the Pteropodidae family, and hemiplasy may also explain the patterns of evolutionary similarity observed in these bats.

## Evolutionary rates analyses

Phylogenetic analyses may also provide information regarding *Evolutionary Rates* (ER), which are very useful to evaluate molecular bases associated with the repeated evolution of similar phenotypes. ERs are estimated from the amount of nucleotide or amino acid changes in a given lineage over a specific period of time (Baum and Smith, 2013). Phenotypic transitions may involve changes in selection forces on the genes or proteins related to those phenotypes, causing a shift in the evolutionary rates of the sequences (Kowalczyk *et al.*, 2019). One approach often used consists of investigating shifts in the ER occurring independently on the branches of lineages with recurrent phenotypes (Partha *et al.*, 2017; Kowalczyk *et al.*, 2019). The branch lengths are calculated for each gene, so these rates are gene-specific, termed as Relative Evolutionary Rates (RER) by Kowalczyk *et al.* (2019). These RER for each gene are then correlated with the evolution of a recurrent phenotype across the phylogeny (Partha *et al.*, 2017; Kowalczyk *et al.*, 2019). 

As aforementioned, topologies corresponding to gene trees may encompass homoplasy, an effect detected by conflicts between gene trees and species trees. Topology differences may also derive from other factors, including gene evolutionary rates. Genes that evolve rapidly are more prone to involve conflicts attributed to ILS (incomplete lineage sorting), which may result in discrepancies between gene and species trees (Degnan and Rosenberg, 2006), especially if estimated lengths of internal branches are shorter in the species tree than in gene trees (Guerrero and Hahn, 2018). Branch lengths may differ between the gene tree and the species tree even in identical topologies (Edwards, 2009). The positioning of tips associated with long branches may also be imprecise due to an artifact named ‘long branch attraction’ (Degnan and Rosenberg, 2009). Estimates of the “hemiplasy risk factor” - given by the ratio between homoplasy and hemiplasy - can be a valuable tool to estimate the likelihood of incongruence resulting from homoplasy or hemiplasy (Guerrero and Hahn, 2018). Ignoring the mismatch between gene and species trees may result in incorrect estimates of substitution rates when mapping sequences from conflicting loci in the species tree (Mendes *et al.*, 2016). To overcome such a challenge, some programs consider gene tree heterogeneity in their approach (Guerrero and Hahn, 2018; Yan *et al.*, 2022). Despite the vast majority of models treating phenotypes as binary, there are some models that consider associations between genomic substitution rates and continuous phenotypes in the analyses implemented (see Kowalczyk *et al.*, 2019). 

Another approach using estimates of ER consists of traditional methods of selection tests hypotheses. These methods are based on codons and therefore useful for coding sequences, and include site (Massingham and Goldman, 2005; Yang *et al.*, 2000), branch (Yang and Nielsen, 2002), branch-site (Zhang *et al.*, 2005) and clade (Yang and Nielsen, 2002; Bielawski and Yang, 2004) models (see Huerta-Cepas *et al.*, 2016; Gao *et al.*, 2019). However, the model that takes into account only the changes among sites (site model) has little utility for analysis of recurrent phenotype. This approach can be used in only one lineage, with a specific trait or set of traits, but may also be implemented to evaluate recurrent phenotypes. Since phenotypic changes are often explained by positive selection, these methods are able to evaluate whether branches or clades with recurring phenotypes likely involve changes in selection regimes (Yang, 1998). 

These analyses usually compare the likelihood of neutral models (which reflect genetic drift, for example) with alternative models of evolution, according to which sequence patterns would reflect adaptive evolution or scenarios of constrained changes (Yang, 2007; Smith *et al.*, 2015). A key variable for these selection tests is the ω value (an indicator of selective pressure), which corresponds to the ratio between nonsynonymous [dN] and synonymous [dS] substitution rates (Nei and Gojobori, 1986; Li, 1993, Yang and Nielsen, 2000). In the branch and clade models, the software compare the *one-ω ratio model*, which assumes the same ω values for all branches, and the *two (or more)-ω ratio model*, which admits different ω values for some pre-established lineages (Yang, 2002). The ω indicates the type of selection regime acting on a protein-coding gene (ω < 1: purifying selection; ω = 1: neutral evolution; and, ω > 1: positive selection; see Zhang *et al.*, 2005; Yang, 2007). The branch-site model approach combines different ratios across sites and across branches (Zhang *et al.*, 2005). In addition to detecting episodic selection along pre-specified branches in the tree, this analysis identifies the sites of a coding gene evolving under purifying, neutral or positive selection (Zhang *et al.*, 2005; Gharib and Robinson-Rechavi, 2013). It should be taken into account, however, that the analysis considering distantly related species can be misinterpreted due to saturation of sites or amino acids (Lamichhaney *et al.*, 2019).

### An example of analyses based on evolutionary rates: Repeated evolution of aquatic mammals

The transition of mammalian lineages to aquatic environments occurred several times and evolved similar phenotypic traits associated to the aquatic lifestyle, including modifications in the hindlimb configuration (Fish and Hui, 1991; Fish *et al.*, 2008), body elongation, and changes in the nostrils relative positioning (Uhen, 2007). Some previous studies have used the ER approach to identify shifts in evolutionary rates among dozens or hundreds of genes (Chikina *et al.*, 2016; Nery *et al.*, 2016), providing evidence for parallel evolution in the evolutionary rates of hundreds of genes during the evolution of three marine mammalian lineages (Cetacea, Pinnipedia and Sirenia; see Chikina *et al.*, 2016). Analyses using selection tests that focused on evolution of *Hox* genes, a family of genes which encodes transcription factors related to the body plans and development (Carroll, 1995), identified that each aquatic mammalian lineage encompasses a different set of positively-selected *Hox* genes, which remarkably overlap in their functions during the development of some of these phenotypic traits (Nery *et al.*, 2016).

### Ancestral sequence reconstruction (ASR)

Ancestral sequence reconstructions (ASR) are used to statistically infer the ancestral sequences of genes, non-coding regions or proteins within the nodes of a given phylogenetic tree, using present-days homologous sequences (Thornton, 2004; Merkl and Sterner, 2016). These methods are useful for studies of recurrent evolution of similar phenotypes, and allow distinguishing nucleotide or amino acid changes as representing convergent or parallel evolution. In this approach, homologous sequences are aligned and each site or amino acid has its evolutionary history reconstructed using a species phylogeny through a variety of software (Table 1). While this approach can be used to study both coding and regulatory sequences, it is particularly advantageous to evaluate mutations occurring at the same site, as the comparisons are performed site-by-site.

The computational methods of ASR use approaches that were originally developed for phylogenetic analyses (Gumulya and Gillam, 2017). The first method used was maximum parsimony (Fitch, 1971), which assumes to be more likely a reconstruction encompassing the minimum number of substitutions. Development of these methods was followed by the advance of probabilistic approaches - maximum likelihood (‘ML’, Yang *et al.*, 1995; Koshi and Goldstein, 1996) and Bayesian reconstructions (Ronquist and Huelsenbeck, 2003; see Gumulya and Gillam, 2017 for a review). The probabilistic methods deal better with unequal ER (what is expected in cases of recurrent phenotypes) and to estimate the confidence of each inferred ancestral state (Gumulya and Gillam, 2017). The Maximum Likelihood methods are classified in two categories: marginal and joint (Yang *et al.*, 1995). The joint reconstruction is considered more suitable for studies of phenotypic recurrence because it adequately accounts for changes in each site, while the marginal reconstruction is preferred for studies aiming to evaluate the molecular sequences in a particular node, being more often used in studies that aim to reconstruct ancestral proteins (Yang *et al.*, 1995; Gumulya and Gillam, 2017).

### Examples of ASR: 1) Ribs in the posterior trunk region of snakes, caecilians and manatees, 2) Resistance to toxic effects of bufadienolides in snakes, 3) Bamboo-eating pandas, 4) Hemoglobin-Oxygen affinity in hummingbirds

Good examples of how ASR analyses contribute to evaluating the repeated evolution of similar phenotypes are illustrated by studies conducted with several animal taxa. The first example we provide relates to the development of ribs in the posterior trunk region (i.e., lumbar region) in some amniote lineages. Most vertebrates exhibit morphologically-distinct regions along the axial skeleton, being the lumbar region characterized by the absence of ribs (Wellik and Capecchi, 2003; Carapuço *et al.*, 2005; McIntyre *et al.*, 2007). Some lineages, however, have ribs associated to the vertebrae in the posterior trunk region - this is the case of iconic animals such as the manatees and elephants (mammals), the snakes (reptiles), and the caecilians (amphibians). The genetic mechanism associated to this rib-associated lumbar morphotype is a recurrent polymorphism that evolved in lineages as distant as snakes, ​​Afrotheria mammals and the lissamphibians Gymnophiona and Urodela (Guerreiro *et al.*, 2013; Pereira *et al.*, 2022). This nucleotide change occurred in the H1 enhancer, a region that regulates the expression of *MYF5*, a gene involved in rib development in vertebrate embryos. An ancestral sequence reconstruction analysis demonstrated that this is an example of parallelism, with the three substitutions identified occurring from the same base T to the same nucleotide C (Pereira *et al.*, 2022).

Another good example of the ASR approach is provided by studies evaluating snake lineages that are resistant to toxic steroids named bufadienolides. These steroids bind to cell membranes and disable the Na+/K+-ATPase pumps, but some predators evolved resistance to these chemical defenses of toads involving toxic steroids bufadienolides (Mohammadi *et al.*, 2016). Toxic resistance apparently evolved in association with mutations observed even in species that do not appear to prey frogs often, and have originated multiple times in predatory lineages (Mohammadi *et al.*, 2016). Two parallel amino acid changes in the H1-H2 extracellular loop of the Na+/K+-ATPase apparently explain the toxin resistance observed in snakes (Mohammadi *et al.*, 2016), being one the Q[Glutamine]111L[Leucine], and the other a G[Glycine]120R[Arginine]. 

As another example of ASR analyses, we also can cite the study of repeated diet transitions to bamboo-eating in carnivores. Two non-phylogenetically related species, the giant panda (*Ailuropoda melanoleuca*, Carnivora, Ursidae) and the red panda (*Ailurus fulgens*, Carnivora, Ailuridae), evolved diets specialized in bamboos and an adaptive pseudothumb (Hu *et al.*, 2017). Signs of adaptive changes in the genes *dync2h1* and *pcnt*, probably involved in the development of a pseudothumb, have been identified from ancestral reconstructions of protein sequences implemented using thousands of orthologs (Hu *et al.,* 2017). From these analyses, the authors proposed two parallelisms in the *dync2h1* gene: R3128K[Lysine] (in giant and red pandas) and K3999R (in giant and red pandas and also in the Weddell seals and walrus). Moreover, the analyses indicate a possible pseudogenization of the umami taste receptor gene *tas1r1* in both panda lineages (Hu *et al.,* 2017).

Finally, variation in the Hemoglobin-Oxygen affinity in birds provides a fourth example of ASR analyses applied to the study of recurrent phenotypes. The Hemoglobin-Oxygen affinity varies according to the atmospheric partial pressure, and animals with high levels of aerobic activity under hypoxic conditions often have optimizing blood-O_2_ affinity (Projecto-Garcia *et al.*, 2013). In South American hummingbirds, colonization of new elevation zones occurred in association with similar amino acid substitutions that changed the respiratory properties of hemoglobin (Projecto-Garcia *et al.*, 2013). Ancestral reconstruction of such changes provide evidence for two parallel amino acid substitutions: G13S[Serine] and G83S (Projecto-Garcia *et al.*, 2013). 

## Repeated trait loss: How ‘absence’ evolved multiple times, and why it challenges strict definitions of convergence and parallelism

Disjunctive expression of phenotypic traits is developmentally feasible, especially when trait expression is settled on switch-regulated mechanisms (see West-Eberhard, 2003). Repeated loss of specific phenotypic traits is very common in evolution, and defies strict definitions of convergence and parallelism because modifications in different components of a signaling pathway may silence developmental processes and result in the absence of that trait in a given lineage. Repeated loss is particularly likely if the structure being lost has some developmental and functional independence from other traits and, therefore, is less subjected to pleiotropic trade-offs (Womack *et al.*, 2018). Phenotypic traits are established during development through intricate signaling pathways encompassing several genes that interact with each other. Accordingly, changes in either component of these signaling cascades might silence the developmental pathway, resulting in the absence of that given trait in the lineage. Given the strict definitions of convergence and parallelism (see Figures 2 and 3), one may ask how to classify changes settled on different components of a given developmental pathway (see West-Eberhard, 2003 for a review). 

An emblematic example of repeated trait loss refers to the multiple origins of snakelike phenotypes in Tetrapoda. Snakelike phenotypes are characterized by elongated bodies and reduced or absent limbs, and entirely limbless species are observed in clades as distant as Lissamphibia and Lepidosauria (see Woltering, 2012). Several studies aimed to identify the genetic bases associated with limb loss in specific groups (e.g. Singarete *et al.*, 2015; Guerreiro *et al.*, 2016; Kvon *et al.*, 2016; Leal and Cohn, 2016; Ovchinnikov *et al.*, 2022; Roscito *et al.*, 2022; also reviewed in Leal and Cohn, 2018), and comparisons among clades provide evidence that different changes in developmental pathways may independently produce the same phenotype characterized by absence of limbs. For example, molecular evolution analyses in three limbless lineages - snakes, amphisbaenians and caecilians - suggest five sites in the first exon of the gene *Hoxa13* evolving under positive selection in snakes (Kohlsdorf *et al.*, 2008), a pattern not identified for this gene in amphisbaenians and caecilians (Singarete *et al.*, 2015). On the other hand, limb loss in snakes and caecilians also seems related to deletions in the ZRS limb-specific enhancer (Kvon *et al.*, 2016; Ovchinnikov *et al.*, 2022). This enhancer regulates the expression of *sonic hedgehog* in developing limbs, a gene that modulates the production of the *SHH* morphogen in the zone of polarizing activity (ZPA), playing a key role in the establishment of the anterior-posterior axis in developing limbs (Petit *et al.*, 2017; Jin *et al.*, 2019). Snakes that are completely limbless (i.e. without vestigial limbs) exhibit a 17-base deletion in ZRS and accelerated evolutionary rates in the sequence of this enhancer (Kvon *et al.*, 2016). This deletion and the high evolutionary rates of the snake ZRS suggest impairment of the enhancer function with consequent relaxed selection, which was confirmed by experiments inserting the snake ZRS into mice that generated individuals with severe limb reduction (Kvon *et al.*, 2016). In caecilians, the ZRS enhancer element seems to be entirely absent from the genomes sequenced so far (Ovchinnikov *et al.*, 2022), suggesting a similar mechanism involved in limb loss in Lissamphibia. However, other limbless squamate species do not exhibit such deletion in the ZRS (Roscito *et al.*, 2022), and the ZRS patterns differ even among closely-related lizard species that exhibit limb reduction and digit loss (Kohlsdorf, 2021), suggesting that the phenotype of absent limbs might also evolve through changes in other genes or cis-regulatory elements.

Another example of recurrent loss is observed in fossorial mammals that spend most of their lives under the surface. Adaptation of the subterranean lifestyle usually involves eye reduction or loss and impairment of the sense of sight (Partha *et al.*, 2017). Recent studies identified accelerated evolutionary rates in genes and enhancers related to eyes in non-phylogenetically related subterranean lineages of moles and mole-rats (lens intrinsic membrane protein 2 [*lim2*] and retinal proteins: retinal outer segment membrane protein 1 [*rom1*] and rod cell-specific G protein, subunit alpha [*gnat1*]) suggesting an intricate mechanism associated to the loss of visual function in these animals (Partha *et al.*, 2017).

These examples illustrate how repeated trait loss defies the identification of developmental changes underlying the absence of a given phenotypic feature in different lineages, especially in studies aiming to classify the associated genetic patterns as *convergence* or *parallelism*. Trait loss involves two complicating aspects for such studies: 1) part of the sequence variation associated to a silenced pathway may correspond to degeneration of the signaling cascade, instead of the mechanism ‘responsible’ for switching off the developmental process; 2) part of sequence conservation observed in a silenced pathway may indicate molecular stability due to pleiotropy. This discussion could be significantly expanded by novel studies considering complete signaling networks, instead of focusing on candidate genes, combined with conceptual discussions addressing the developmental processes underlying a disjunct expression of phenotypic traits along the phylogeny.

Research in the past decade produced a considerable number of studies addressing the processes and mechanisms related to the repeated evolution of similar phenotypes, which nurtured discussions about homoplasy and encouraged reexamination of key concepts, including convergence and parallelism. In this review, we use an integrated approach to discuss this topic, which consists of revisiting the classical definitions of convergence and parallelism, describing some comparative methods used to assess the evolution of repeated phenotypes, and examining how repeated trait loss challenges strict definitions of convergence and parallelism. To illustrate how different methodological approaches can be used to evaluate such evolutionary patterns, we provide examples of studies focusing on various lineages. A major goal of this review is to highlight the importance of combining modern analytical phylogenetic tools with knowledge about developmental pathways and regulatory mechanisms to completely understand the repeated evolution of similar phenotypes. Despite challenges for the study of developmental pathways in biological systems that are not experimental models, the growing number of genomes available and the proliferation of analytical tools designed to operate large amounts of data stimulate significant progress in the field. As the depth of knowledge increases, so does its ability to reveal the genetic and molecular mechanisms enabling recurrent evolution in biological lineages.
